# Serum Phosphatidylcholine Species 32:0 as a Biomarker for Liver Cirrhosis Pre- and Post-Hepatitis C Virus Clearance

**DOI:** 10.3390/ijms25158161

**Published:** 2024-07-26

**Authors:** Kilian Weigand, Georg Peschel, Jonathan Grimm, Marcus Höring, Sabrina Krautbauer, Gerhard Liebisch, Martina Müller, Christa Buechler

**Affiliations:** 1 Department of Internal Medicine I, Gastroenterology, Hepatology, Endocrinology, Rheumatology, and Infectious Diseases, University Hospital Regensburg, 93053 Regensburg, Germany; kilian.weigand@gk.de (K.W.); georg.peschel@klinikum-ffb.de (G.P.); jonathan.grimm@stud.uni-regensburg.de (J.G.); martina.mueller-schilling@klinik.uni-regensburg.de (M.M.); 2 Department of Gastroenterology, Gemeinschaftsklinikum Mittelrhein, 56073 Koblenz, Germany; 3 Department of Internal Medicine, Klinikum Fürstenfeldbruck, 82256 Fürstenfeldbruck, Germany; 4 Institute of Clinical Chemistry and Laboratory Medicine, University Hospital Regensburg, 93053 Regensburg, Germany; marcus.hoering@klinik.uni-regensbug.de (M.H.); sabrina.krautbauer@klinik.uni-regensburg.de (S.K.); gerhard.liebisch@klinik.uni-regensbug.de (G.L.)

**Keywords:** hepatitis C, direct-acting antivirals, liver cirrhosis, genotype, phosphatidylcholine

## Abstract

Phosphatidylcholine (PC) is an essential lipid for liver health and lipoprotein metabolism, but its circulating levels have rarely been studied in patients with cirrhosis. Chronic hepatitis C virus (HCV) infection causes lipid abnormalities and is a major cause of cirrhosis. Effective HCV elimination with direct-acting antivirals (DAAs) is associated with the normalization of serum low-density lipoprotein cholesterol levels. Since PC is abundant in all lipoprotein particles, this study analyzed the association between serum PC species levels and liver cirrhosis before and after HCV eradication. Therefore, 27 PC species were measured by Fourier Transform Mass Spectrometry in the serum of 178 patients with chronic HCV infection at baseline and in 176 of these patients at the end of therapy. The PC species did not correlate with viral load, and the levels of 13 PC species were reduced in patients infected with genotype 3a compared to those affected with genotype 1. Four PC species were slightly elevated 12 weeks after DAA initiation, and genotype-related changes were largely normalized. Patients with HCV and cirrhosis had higher serum levels of PC 30:0 and 32:0 before and at the end of therapy. PC species containing polyunsaturated fatty acids were mostly decreased in cirrhosis. The levels of polyunsaturated, but not saturated, PC species were inversely correlated with the model of the end-stage liver disease score. A receiver operating characteristic curve analysis showed area under the curve values of 0.814 and 0.826 for PC 32:0 and 0.917 and 0.914 for % PC 32:0 (relative to the total PC levels) for the classification of cirrhosis at baseline and at the end of therapy, respectively. In conclusion, the specific upregulation of PC 32:0 in cirrhosis before and after therapy may be of diagnostic value in HCV-related cirrhosis.

## 1. Introduction

Chronic hepatitis C virus (HCV) infection is a common cause of liver inflammation, which triggers fibrosis and finally liver cirrhosis [1]. HCV infection is closely linked with patients’ lipid metabolism, and the virus depends on the lipoprotein machinery of the host for a large part of its life cycle [2,3].

Direct-acting antivirals (DAAs) eliminate HCV within weeks and can achieve a sustained virological response (SVR) of up to 100% [4]. The eradication of HCV is related to the normalization of serum cholesterol as well as low-density lipoprotein (LDL) levels, which decline in HCV infection [5,6,7,8]. LDL carries lipid classes, such as cholesteryl esters, lysophosphatidylcholines, sphingolipids and phosphatidylcholines (PC), and PCs make up about 30% of LDL lipids [9]. PC is essential for the release of very-low-density lipoprotein. The retention of PC in the cell or impaired PC synthesis due to choline deficiency contributes to liver steatosis [10,11].

PC is the most abundant glycerophospholipid in the membranes of mammalian cells and represents 40–50% of total phospholipids [12]. The chain length and degree of saturation of the fatty acyl moieties in PC exhibit significant diversity, which determines the biophysical characteristics of cell membranes. This regulates membrane-associated processes, such as receptor localization and signal transduction [12,13].

Acyl-chain incorporation occurs during the de novo synthesis of precursor phosphatidic acid from lysophosphatidic acid by lysophosphatidic acid acyltransferases. It also happens during fatty acid remodeling, catalyzed by lysophosphatidylcholine acyltransferases (LPCATs) [12,13].

It was shown that HCV-infected hepatocytes have a low expression of LPCAT1, an enzyme which converts lysophosphatidylcholine to phosphatidylcholine [14]. Low cellular LPCAT1 levels were related to a higher production of infectious HCV particles, suggesting a role of reduced PC in HCV severity [14]. Accordingly, the PC and PE levels in the liver tissue of patients with HCV were low in comparison to patients with hepatitis B virus (HBV), and the polyunsaturated variants of these lipid classes were especially reduced [15].

However, in Huh7.5 cells with chronic HCV infection, the blockage of choline uptake for PC synthesis reduced the production of infectious virus particles [16]. It was also observed that there was about three-fold increased PC in the endoplasmic reticulum of HCV-infected cells, which is likely caused by the upregulation of phosphatidylethanolamine (PE) transferase (PEMT), which converts PE to PC [17].

The PEMT pathway contributes about 30% of liver PC, while the cytidine diphosphate (CDP)–choline pathway accounts for about 70%. Choline kinase alpha catalyzes the reaction between choline and adenosine triphosphate, producing adenosine diphosphate and phosphocholine, an intermediate in the CDP–choline pathway. This enzyme has been shown to increase HCV replication [18]. The knockdown of CTP cytidyltransferase alpha, the rate-limiting enzyme in the CDP–choline pathway, had no effect on HCV replication, and the cellular PC levels remained normal in cells with inactive choline kinase alpha [18]. Therefore, the involvement of cellular PC in HCV infection remains unclear.

Chronic HCV infection is a major cause of liver cirrhosis, which is associated with extreme lipid abnormalities. In patients with liver cirrhosis LDL, high-density lipoprotein (HDL) and lipids carried by these lipoproteins are reduced [9,19,20,21,22,23].

PC is very abundant in circulation, far exceeding the levels of lysophosphatidylcholine and sphingolipids [9]. However, the circulating PC levels do not decline in patients with liver cirrhosis, which is associated with low levels of different lipid classes such as cholesterol and ceramides [23,24,25,26]. A few PC species were associated with the decompensation of cirrhosis and were further decreased in patients who did not survive [27]. An analysis of serum from patients with chronic HBV showed that polyunsaturated PCs were downregulated in cirrhosis compared to patients with chronic HBV and to liver-healthy controls [28]. However, in the plasma of patients co-infected with human immunodeficiency virus/HCV, PC species were not related to liver cirrhosis. Notably, at SVR, an oxidized PC species suggested to exert anti-inflammatory effects was increased [29].

The serum LDL and cholesterol levels are elevated early post-initiation of DAA therapy and is consistent with low levels in HCV infection [30]. However, the impacts of HCV infection and DAA therapy on circulating PC levels remain largely unknown. PC species vary in acyl chain length and number of double bonds, which impact their biological activity [9,12,13], but they have rarely been studied in HCV infection and liver cirrhosis.

Non-invasive tests for the diagnosis of liver fibrosis have been developed, but recent studies have shown that these analyses reflect fibrosis and inflammation and decline accordingly during HCV eradication, with no improvement in the liver fibrosis scores [31,32,33]. Our main aim was to investigate the serum circulating PC species levels before and after HCV cure to identify associations with liver disease severity.

## 2. Results

### 2.1. Serum PC Species Levels in Relation to Gender, Age, Body Mass Index, Liver Steatosis and Diabetes

Twenty-seven PC species were measured in the serum of 178 patients with chronic HCV before starting DAA therapy. The cohort is described in Table 1. Serum from these patients was available 4 weeks after the start of therapy. For 176 of these patients, serum was also available at the end of DAA therapy 12 weeks later (Table 1). At the end of therapy, ferritin, alanine aminotransferase (ALT) and aspartate aminotransferase (AST) were lower, and LDL was increased (Table 1).

**Figure 1:**
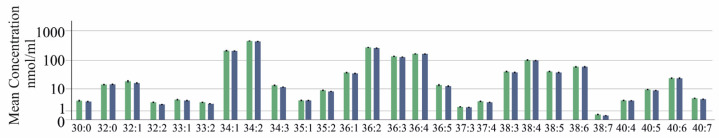
PC species in serum of female (green bars) and male (blue bars) patients with HCV before therapy. Mean concentration ± standard deviation are shown.

The levels of the 27 PC species of the 74 female and the 104 male patients were similar (Figure 1). These PC species did not correlate with the BMI (*p* > 0.05 for all). Age was positively associated with PC 30:0 (r = 0.320, *p* < 0.001), PC 32:0 (r = 0.388, *p* < 0.001) and PC 33:1 (r = 0.251, *p* = 0.02). The patients with (74 patients) and without (104 patients) ultrasound-diagnosed liver steatosis had similar serum PC species levels (*p* > 0.05 for all). The 20 patients with diabetes had higher PC 32:0 (*p* = 0.053), whereas the remaining 26 PC species were comparable to non-diabetic patients.

### 2.2. PC Species in Relation to Liver Fibrosis

**Figure 2:**
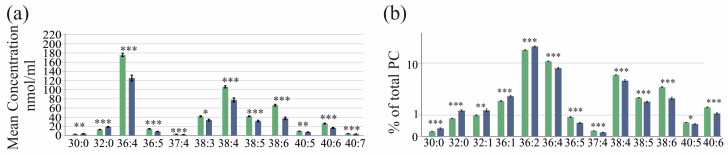
PC species in serum of patients with HCV before DAA therapy with respect to liver cirrhosis diagnosed by ultrasound. For clarity, only the PC species which differed between both cohorts are shown. (**a**) PC species in serum of patients without (green bars) and with (blue bars) liver cirrhosis; (**b**) PC species relative to total PC levels in % in serum of patients without (green bars) and with (blue bars) liver cirrhosis. Data are displayed in logarithmic scale for better visualization of low abundant PC species. * *p* < 0.05, ** *p* < 0.01 and *** *p* < 0.001. Statistical test used: Mann–Whitney U-test. Mean values ± standard deviations are shown.

In liver cirrhosis, the serum lipoprotein levels are low [34]. The patients with HCV with liver cirrhosis diagnosed by ultrasound (40 patients) had reduced serum levels of PC 36:4, 36:5, 37:4, 38:3, 38:4, 38:5, 38:6, 40:5, 40:6 and 40:7 compared to the patients with HCV without liver cirrhosis. The PC 30:0 and PC 32:0 levels were increased (Figure 2a). The total serum PC levels were not decreased in cirrhosis (*p* > 0.05).

Patients with liver cirrhosis were older, and this may explain the higher PC 30:0 and 32:0 levels, which correlated with age (please see above). When an analysis was performed in patients aged above 50 years, age (*p* = 0.095) and PC 30:0 (*p* < 0.05) did not differ between the 35 patients with cirrhosis and the 72 patients without cirrhosis, whereas PC 32:0 was still significantly increased in cirrhosis (*p* < 0.001).

The PC species profile of patients with ultrasound-diagnosed liver cirrhosis and those without cirrhosis was quite different. The percentages of PC 30:0, 32:0, 32:1, 36:1 and 36:2 (relative to total PC concentration) were higher, and the % of PC 36:4, 36:5, 37:4, 38:4, 38:5, 38:6, 40:5 and 40:6 were reduced in the patients with cirrhosis versus the patients without cirrhosis (Figure 2b).

Diabetes is a common comorbidity of cirrhosis [35], but the 12 patients with diabetes with liver cirrhosis had similar PC 32:0 (*p* = 1.000) and % PC 32:0 levels (*p* = 0.942) compared to the patients with cirrhosis without diabetes.

**Figure 3:**
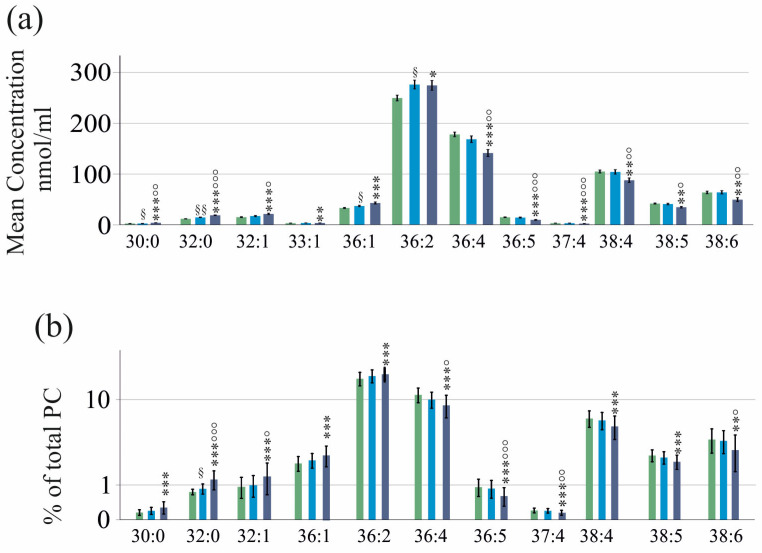
PC species in the serum of patients with HCV before DAA therapy in relation to the FIB-4 score. For clarity, only the PC species which differed between both cohorts are shown. (**a**) PC species in the serum of patients with no fibrosis (green bars) with an indeterminate FIB-4 score (light blue bars) and patients with advanced fibrosis (blue bars); (**b**) % of PC species in the serum of the patients described in a. The data are displayed in a logarithmic scale for better visualization of low abundant PC species. * *p* < 0.05, ** *p* < 0.01 and *** *p* < 0.001 for the comparison of patients with no fibrosis and patients with definite fibrosis; ^°^ *p* < 0.05, ^°°^ *p* < 0.01 and ^°°°^ *p* < 0.001 for the comparison of patients with advanced fibrosis and patients with an intermediate FIB-4 score. ^§^ *p* < 0.05 and ^§§^ *p* < 0.01 for the comparison of patients with no fibrosis and patients with an intermediate FIB-4 score. Statistical test used: Kruskal–Wallis-test. Mean concentrations ± standard deviations are shown.

The fibrosis-4 (FIB-4) score is a non-invasive measure for the diagnosis of liver fibrosis [36]. Patients with fibrosis had higher levels of PC 30:0, 32:0, 32:1, 33:1, 36:1 and 36:2 and lower levels of PC 36:4, 36:5, 37:4, 38:4, 38:5 and 38:6 than patients without fibrosis. The PC 30:0, 32:0 and 32:1 levels of patients with severe fibrosis were higher, and the PC 36:4, 36:5, 37:4, 38:4, 38:5 and 38:6 levels were reduced compared to the patients with indeterminate FIB-4 scores. PC 30:0, 32:0, 36:1 and 36:2 differed between patients with no fibrosis and those with an indeterminate FIB-4 score (Figure 3a).

Biomarkers for diagnosing moderate fibrosis are still needed [37]. These PC species were tested for their suitability in identifying such patients. The area under the receiver operating curve (AUROC) for the discrimination of no fibrosis from indeterminate fibrosis was 0.681 for PC 32:0 (*p* = 0.013). PC 30:0, 36:1 and 36:2 could not separate these groups (*p* > 0.05). The total serum PC levels did not change with an increasing FIB-4 score (*p* > 0.05).

The percentages of PC 30:0, 32:0, 32:1, 36:1 and 36:2 were higher, and the % of PC 36:4, 36:5, 37:4, 38:4, 38:5 and 38:6 were lower for patients with severe fibrosis compared to patients without fibrosis. The percentages of PC 32:0 and 32:1 of patients with definite fibrosis were higher compared to those of patients with indeterminate scores. The relative levels of PC 36:4, 36:5, 37:4 and 38:6 of patients with severe fibrosis were reduced in comparison to patients with indeterminate scores (Figure 3b). The percentage of PC 32:0 also differed between patients with no fibrosis and those with an intermediate FIB-4 score, and the AUROC of % PC 32:0 was 0.711 (*p* = 0.001).

### 2.3. PC Species in Relation to Laboratory Measures of Hepatic and Renal Function

Since several PC species differed in the serum of patients with and without cirrhosis (Figure 2a), the correlations of PC species with laboratory measures were separately calculated in these groups.

In patients without liver cirrhosis, PC species were mostly not correlated with AST, ALT, the international normalized ratio (INR), bilirubin, albumin, the MELD score or creatinine. Here, only PC 32:0 (r = 0.405, *p* < 0.001) and PC 36:1 (r = 0.281, *p* = 0.022) were positively correlated with AST.

In the subgroup of patients with liver cirrhosis, PC 32:0 was positively related with bilirubin (r = 0.511, *p* = 0.020), and PC 37:4 (r = 0.545, *p* = 0.007), 38:6 (r = 0.677, *p* < 0.001) and PC 40:6 (r = 0.548, *p* = 0.007) with albumin. PC 36:4 (r = −0.502, *p* = 0.007), 37:4 (r = −0.593, *p* = 0.002), 38:3 (r = −0.553, *p* = 0.006), 38:4 (r = −0.544, *p* = 0.008), 38:6 (r = −0.623, *p* < 0.001), 40:6 (r = −0.615, *p* < 0.001) and 40:7 (r = −0.488, *p* = 0.038) were negatively correlated with INR. PC 38:6 (r = −0.509. *p* = 0.022) and PC 40:6 (r = −0.499, *p* = 0.028) were negatively correlated with the MELD score.

### 2.4. PC Species in Relation to Markers of Inflammation and Thrombocyte Count

In patients without liver cirrhosis, PC 32:0 was negatively correlated with the platelet count (r = −0.356, *p* < 0.001). PC 40:7 was negatively associated with the leukocyte count (r = −0.293, *p* = 0.013). PC species were not related to CRP. In liver cirrhosis, PC species were not related with CRP, the leukocyte count or platelets (*p* > 0.05 for all).

### 2.5. PC Species in Relation to Viral Load and Viral Genotype

The viral load was not related with the serum PC species levels (*p* > 0.05 for all). In patients without liver cirrhosis, 43 patients were infected with genotype 1a, 53 patients with genotype 1b, 29 patients with genotype 3a and 13 patients with infrequent HCV genotypes, which were assembled in one group.

**Figure 4:**
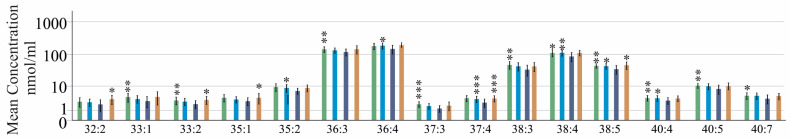
PC species in serum of patients with HCV in relation to genotype. PC species, which are altered by genotype, are shown in serum of patients with 1a (green bars), 1b (light blue bars), 3a (blue bars) and rare genotypes (orange bars) * *p* < 0.05, ** *p* < 0.01 and *** *p* < 0.001 for comparison with genotype 3a. Statistical test used: Kruskal–Wallis-test. Mean concentrations ± standard deviations are shown.

PC 33:1, 33:2, 36:3, 37:3, 38:3, 38:4, 38:5, 40:4, 40:5 and 40:7 were low in genotype 3a compared to genotype 1a. PC 35:2, 36:4, 37:4, 38:5 and 40:4 were low in 3a compared to 1b. PC 33:2, 37:4 and 38:5 were lower in 3a than patients with rare genotypes (Figure 4). The percentages of PC 37:3, 37:4 and 38:3 were higher in 1a than 3a (*p* < 0.001).

### 2.6. PC Species in Relation to Viral Cure

**Figure 5:**
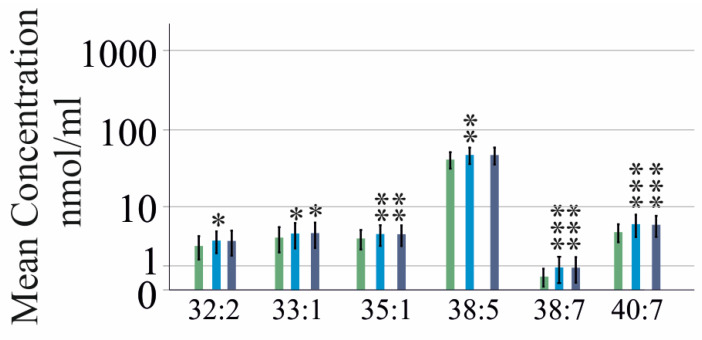
PC species changed in serum of patients with HCV during DAA therapy. Patients with liver cirrhosis were excluded. Serum PC species before therapy (green bars), at 4 weeks (light blue bars) and at 12 weeks (blue bars) after start of therapy. Data are shown in logarithmic scale for better visualization of low abundant PC species. * *p* < 0.05, ** *p* < 0.01 and *** *p* < 0.001 for comparison of PC levels at 4 and/or 12 weeks after therapy start compared to pretreatment levels. Statistical test used: Friedman test.

In patients without cirrhosis, the PC 33:1, 35:1, 38:7 and 40:7 levels increased at 4 and 12 weeks after starting therapy compared to the pretreatment levels. The PC 32:2 and 38:5 levels were elevated at 4 weeks but not at 12 weeks (Figure 5). The percentages of total PC for 34:3, 38:5, 38:7 and 40:7 increased, while PC 36:2 decreased at the end of therapy.

In patients with cirrhosis, the PC 36:5 levels increased at 4 and 12 weeks post-therapy initiation compared to the pretreatment levels. PC 34:1 was elevated at 4 weeks but not at 12 weeks. The percentages of PC 34:1, 36:5 and 40:7 increased, while that of PC 36:2 decreased at the end of therapy.

Genotype-related differences were mostly resolved after viral clearance. At 12 weeks post-therapy, PC 40:7 (*p* = 0.006) and % PC 40:7 (*p* = 0.009) were higher in genotype 1b compared to 3a, and % PC 38:6 was higher in genotype 1b compared to 1a (*p* = 0.008).

### 2.7. PC Species in Relation to Laboratory Measures Post-DAA Therapy

In our study, all patients achieved SVR12 [7]. At the end of therapy, the patients with HCV with ultrasound-diagnosed liver cirrhosis had reduced PC 36:4, 36:5, 37:4, 38:3, 38:4, 38:5, 38:6, 38:7, 40:6 and 40:7 levels in serum compared to the patients with HCV without liver cirrhosis. PC 30:0 and PC 32:0 were increased. The percentages of PC 36:1, 36:2 and 36:4 were higher in cirrhosis and the % of PC 36:5, 37:4, 38:4, 38:5, 38:6 and 40:6 were lower in cirrhosis.

At the end of treatment, in the non-cirrhosis group, correlations of PC species with the MELD score, bilirubin, INR, ALT, AST and creatinine were not significant.

In the cirrhosis cohort, PC 32:0 was negatively correlated (r = −0.552, *p* = 0.005) and PC 38:4 was positively (r = 0.509, *p* = 0.018) correlated with albumin. PC 38:4 (r = −0.583, *p* = 0.002), PC 38:5 (r = −0.486, *p* = 0.039), PC 38:6 (r = −0.607, *p* = 0.001), PC 40:6 (r = −0.576, *p* = 0.003) and PC 40:7 (r = −0.563, *p* = 0.004) were negatively correlated with INR. PC 37:4 (r = −0.541, *p* = 0.007), PC 38:3 (r = −0.553, *p* = 0.005), 38:4 (r = −0.667, *p* < 0.001), PC 38:6 (r = −0.545, *p* = 0.006), PC 40:5 (r = −0.483, *p* = 0.038) and PC 40:6 (r = −0.576, *p* = 0.002) were negatively correlated with the MELD score.

### 2.8. PC Species to Discriminate Patients with and without Liver Cirrhosis

**Figure 6:**
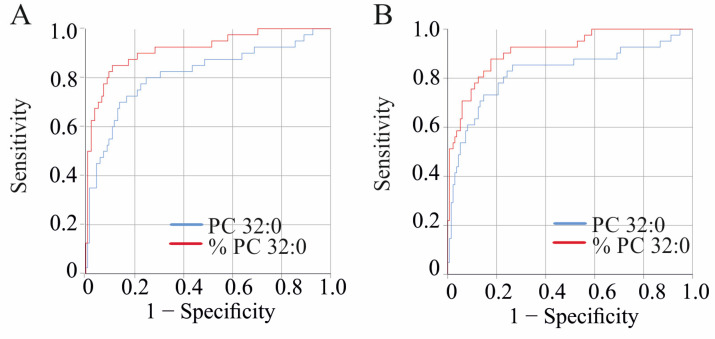
Receiver operating characteristic curve curve for discrimination of patients with HCV with and without liver cirrhosis. (**A**) Before therapy. (**B**) At end of therapy.

The ROC curve analysis identified PC species that could distinguish patients with and without liver cirrhosis diagnosed by ultrasound. PC 32:0 and %PC 32:0 showed AUROC values > 0.8 before and after therapy. Before treatment, the AUROC values were 0.814 for PC 32:0 and 0.917 for %PC 32:0 (*p* < 0.001) (Figure 6a). A concentration of 16.24 nmol/mL PC 32:0 differentiated cirrhosis with 70% sensitivity and 86% specificity, while 0.89% PC 32:0 had 90% sensitivity and 79% specificity.

At the end of therapy, the AUROC values were 0.826 for PC 32:0 and 0.914 for %PC 32:0 (*p* < 0.001) (Figure 6b). A concentration of 17.16 nmol/mL PC 32:0 differentiated cirrhosis with 73% sensitivity and 85% specificity, while 0.92% PC 32:0 had 88% sensitivity and 82% specificity.

The MELD score predicts mortality in cirrhosis and differs between patients with and without liver cirrhosis [38]. In our cohort, the MELD score was similar before and after treatment (Table 1). The AUROC of the MELD score was 0.898 before and 0.877 after DAA treatment (which did not differ, *p* = 0.573), indicating excellent performance to differentiate cirrhosis and non-cirrhosis patients (*p* < 0.001) [39]. Before treatment, the MELD score had 88% sensitivity and 78% specificity for cirrhosis diagnosis. At the end of therapy, the MELD score had 83% sensitivity and 78% specificity for cirrhosis diagnosis.

The AUROCs for PC 32:0 before and after therapy were comparable (*p* = 0.847). Moreover, the AUROC of PC 32:0 and the MELD score before DAA therapy (*p* = 0.0992) and at the end of therapy (*p* = 0.358) were similar.

## 3. Discussion

This study demonstrates that the serum PC 32:0 and %PC 32:0 levels, relative to the total PC levels, distinguish patients with cirrhosis from non-cirrhotic patients. The main results of our study are summarized in Figure S1.

PC is abundant in LDL particles, the systemic levels of which are low in HCV infection and rapidly normalize during DAA therapy [6,8,23,40]. The LDL levels at the end of therapy are approximately 125% of the pretreatment levels, and the increase in PC species ranges from 114 to 200%. Only 4 of the 27 PC species analyzed were higher at the end of therapy compared to the pretreatment levels. PC 32:2 and 38:5 were higher at 4 weeks but not at 12 weeks after the start of therapy. Although the underlying mechanisms cannot be assessed in an observational study such as ours, the current results suggest a complex regulation of serum PC species levels during HCV infection and viral cure.

The serum PC species levels in our patients were not associated with viral load. HCV genotypes 1 and 3 are common genotypes [41], and the majority of our patients were infected with 1a, 1b and 3a. Previous studies have shown that genotype 3 infection affects lipid classes such as ceramides, triglycerides or lathosterol more than genotype 1 infection [23,42,43]. Consistent with this, patients infected with genotype 3a had lower levels of several PC species compared to those infected with 1a or 1b. It is worth noting that the viral genotype did not affect the lysophosphatidylcholine species levels [24], indicating that the reduced conversion of lysophosphatidylcholine to PC is not the underlying mechanism.

The expression of PEMT was found to be over 50% higher in liver biopsies from individuals infected with HCV genotype 3 compared to genotype 1 and three times higher than in individuals with chronic HBV [17]. This study suggests that genotype 3 infection is associated with the accumulation of cellular PC. Our results show low serum PC in these patients, suggesting an impaired release of PC into the circulation, thereby promoting liver steatosis. However, in our cohort, patients with liver steatosis had comparable serum PC levels to those without fatty liver, suggesting that low serum PC levels in genotype 3 infection are not associated with liver fat accumulation.

Compared with patients with HBV, patients with HCV were also found to have reduced levels of hepatic PC, with the most significant reduction observed for the polyunsaturated variants [15]. Current evidence on the hepatic PC levels in HCV is inconclusive and requires further analysis to resolve this issue.

Advanced liver fibrosis in our study cohort was diagnosed by ultrasound and the FIB-4 score. Although the PC species found to be altered in cirrhosis defined by these two approaches were partly discordant, there was an agreement that liver cirrhosis in HCV was associated with higher levels of saturated PC species and a decrease in polyunsaturated PC species. Membrane structural order and membrane lipid packing are increased by saturated fatty acids and decreased by polyunsaturated fatty acids, suggesting that cell function and signal transduction differs between patients with and without cirrhosis [12,44].

Other studies failed to identify associations of circulating PC species levels with liver cirrhosis [26,29], and a decrease in circulating PCs has been described in patients with decompensated liver cirrhosis [27]. It should be noted that all of our patients had compensated cirrhosis. In chronic HBV, polyunsaturated PCs were downregulated, and PC 32:0 was induced in cirrhosis compared to patients with chronic HBV [28]. Although this and our results are not fully concordant regarding the types of unsaturated PC species that decreased in cirrhosis, the observation of an increase in saturated PC and a decrease in unsaturated PC is consistent with our findings.

In cirrhosis, serum PC 32:0 correlated positively with bilirubin and negatively with albumin. Unsaturated PC species showed a positive association with albumin and a negative association with the INR and MELD scores. The PC 32:0 levels and % of PC 32:0 (relative to total PC) effectively distinguished patients with and without cirrhosis both before and after DAA therapy. Patients with cirrhosis were older than those without, and the PC 32:0 levels increased with age. Of clinical relevance, PC 32:0 still differentiated cirrhosis in the age-matched patients. More importantly, preliminary data from our study suggest that PC 32:0 may also discriminate patients without fibrosis from patients with moderate fibrosis as assessed by the FIB-4 score.

The non-invasive analysis of liver fibrosis using sonographic acoustic radiation force impulse has shown improvement in liver stiffness shortly after HCV clearance [33]. The FIB-4 score is calculated using AST and ALT, both of which improve at the end of therapy [45]. This shows that these non-invasive markers are associated with liver inflammation and may not accurately diagnose fibrosis. Therefore, other diagnostic tools not related to HCV-induced inflammation may be more appropriate for assessing liver fibrosis.

The main non-invasive tests for detecting liver fibrosis all have some disadvantages. Transient elastography requires dedicated equipment, and point shear wave elastography gives false positive results in acute hepatitis. The combination of different analyses may improve the diagnosis of fibrosis stages [46]. The FIB-4 score decreases after HCV eradication without an improvement in liver fibrosis [45]. An analysis of PC 32:0 is comparatively inexpensive, but a disadvantage is that this analysis must be performed by specialized facilities and is not yet a validated clinical test.

BMI, diabetes, liver steatosis and sex may confound the analysis of serum lipids related to liver disease severity. However, the 27 measured PC species did not differ between sexes. Diabetes was linked to higher PC 32:0 levels, but this increase was not significant. The PC 32:0 levels were higher in the fasting serum of patients with pre-diabetes compared to healthy controls but did not distinguish patients with type 2 diabetes, who had elevated levels of PC 38:7 and PC 40:6, from healthy controls [47]. Diabetes is a risk factor for metabolic liver steatosis, which was not assessed in this study [47].

Metabolic dysfunction in patients with HCV may result from viral infection and/or metabolic factors that are difficult to distinguish. Our patients with HCV, with and without liver steatosis, had similar PC species levels. In addition, the patients with cirrhosis had comparable PC 32:0 and %PC 32:0 levels regardless of diabetes status. Therefore, serum PC 32:0 can be used as a marker of cirrhosis and possibly fibrosis staging in patients with HCV independent of gender, diabetes and fatty liver. Further research is needed to determine the diagnostic value of measuring serum PC 32:0 in patients with HCV. Since PC 32:0 levels are not related to the MELD score, they may serve as a novel and independent marker of fibrosis/cirrhosis. There are also studies that have shown improvement in the MELD score until post-treatment at week 12 [48,49]. Moreover, the MELD score was not developed to diagnose liver cirrhosis but reflects the mortality of patients on the waiting list for liver transplantation. Therefore, liver cirrhosis should not be diagnosed based on the MELD score [50].

Of clinical relevance is the analysis of PC 32:0 in non-HCV patients. Chen et al. described increased PC 32:0 in patients with HBV-induced cirrhosis, but its suitability as a cirrhosis marker was not evaluated [28]. Hopefully, future studies will address this issue.

The diagnosis of mild fibrosis remains a challenge, and non-invasive biomarkers are needed [37,51]. PC 32:0 and % PC 32:0 could significantly discriminate patients with no fibrosis from those with an intermediate FIB-4 score, suggesting mild/modest fibrosis [36,52]. It is therefore worth evaluating the suitability of this lipid species for the diagnosis and monitoring of fibrosis.

This study has limitations. Healthy controls were not included in the study, and the PC species levels of patients with chronic HCV and the controls were not compared. Fasting has no effect on the plasma PC levels [53], and the serum of our cohort was not collected in the fasting state. However, we cannot exclude the possibility that PC species levels differ between the fed and the fasted state, as has been shown for erythrocyte phospholipid composition [54].

In conclusion, this study demonstrated that serum PC 32:0 is a valuable non-invasive marker for the diagnosis of cirrhosis in chronic HCV and should be evaluated in other chronic liver diseases as a biomarker for cirrhosis and fibrosis staging.

## 4. Materials and Methods

### 4.1. Study Cohort

Serum from patients, who had never been treated for HCV, was collected at the Department of Internal Medicine I (University Hospital of Regensburg) from October 2014 to September 2019. Venous blood was sampled on coagulation activator-coated beads in S-Monovette^®^ Serum CAT tubes (Sarstedt, Nürnbrecht, Germany). The blood was clotted for 20 to 30 min and was centrifuged at 2000 g for 15 min at room temperature. Serum was stored at −80 °C in Eppendorf cups.

All patients were suitable for DAA therapy (sofosbuvir/ledipasvir, glecaprevir/pibrentasvir, sofosbuvir/daclatasvir, elbasvir/grazoprevir or sofosbuvir/velpatasvir) according to the recommendations of the European Association for the Study of the Liver [4]. Patients were over 18 years old. Patients with human immunodeficiency virus or HBV infection and patients with decompensated liver cirrhosis were excluded.

Almost all of our patients were enrolled shortly after the approval of DAA therapies because at that time, adverse effects of statins were expected [55] and statins were discontinued at the start of DAA therapy and restarted after the end of therapy. Pitavastatin therapy for 180 days in patients with dyslipidemia with metabolic syndrome was associated with the normalization of LDL-cholesterol levels and an increase in the PC species levels, including an increase in PC 32:0 by approximately 25% [56]. This may indicate that stopping statins during DAA treatment may cause a decrease in these lipids. None of the PC species decreased during DAA therapy, suggesting a small effect of stopping statins during DAA therapy. Cirrhosis is associated with hypertrophy of the caudate lobe and lateral segment compared to controls. Cirrhosis is also associated with atrophy of the medial segment, anterior segment and right lobe compared with controls [57]. The cut-off values for the FIB-4 scores were as follows: >3.25 for advanced fibrosis, <1.3 for patients with no fibrosis aged less than 65 years, and <2.0 for patients over 65 years [58].

### 4.2. Measurement of PC Species

The analysis of PC was performed by flow injection analysis Fourier Transform Mass Spectrometry (FIA-FTMS) on a hybrid quadrupole-Orbitrap mass spectrometer (Thermo Fisher Scientific, Waltham, MA, USA), and the method was described in detail before [59]. The internal standards PC 14:0/14:0 (1.29 nmol) and PC 22:0/22:0 (1.39 nmol) were added prior to lipid extraction. Ten microliters of serum was extracted using the Bligh and Dyer procedure by Bligh and Dyer [60]. After phase separation, the chloroform phase (containing lipids) was recovered with a pipetting robot (Tecan Genesis RSP 150) and vacuum-dried. The residue was dissolved in chloroform/methanol/2-propanol (1:2:4 *v*/*v*/*v*) in 7.5 mM ammonium formate. PC species were analyzed as [M+HCOO]^−^ in negative ion mode in the m/z range of 520–960 with a maximum injection time of 200 ms, an automated gain control (AGC) of 1 × 10^6^, three microscans and a target resolution of 140,000 (at *m*/*z* 200) [61]. A full-scan mass spectrum of one serum sample is shown in Figure S2. FTMS spectra were processed using the ALEX software [62]. The extracted data were further processed by self-programmed Macros using Microsoft Excel 2016. The quantification was carried out by the multiplication of the spiked IS amount with the analyte-to-IS ratio.

PC 32:0 refers to a PC molecule with two saturated fatty acids. The sum of the acylchain length of both fatty acids is 32, but the exact composition cannot be determined by our approach.

### 4.3. Analysis of Laboratory Values and Calculation of MELD Score

The analysis of laboratory parameters is described in much detail in a recent publication [24]. The MELD is a composite score derived from three laboratory values: the INR, serum total bilirubin and serum creatinine [63].

### 4.4. Statistical Analysis

The mean values of the PC species are shown as boxes ± standard deviation. The Wilcoxon test, Friedman test, Mann–Whitney U-test, Kruskal–Wallis test and ROC analysis were used (SPSS Statistics 26.0 program). Data in tables are given as median, minimum and maximum values. The Shapiro–Wilk test showed normal distributions of PC 36:3, 36:4, 37:4, 38:3, 38:4 and 38:5 before therapy and of PC 36:2, 36:3, 36:4, 38:4, 38:5 and 38:6 at the end of therapy, whereas all other PC species and routine laboratory values were not normally distributed. Therefore, we decided to use non-parametric tests, which can be used for all kinds of data [64], for the analysis of all data. The comparison of two ROC curves was performed with MedCalc version 22.030 (free trial, MedCalc Software Ltd., Ostend, Belgium). The PC data were adjusted for multiple comparisons by multiplying all *p*-values by 27, and a value of *p* < 0.05 was regarded as significant.

## Figures and Tables

**Table 1:** Age, sex, body mass index (BMI) and laboratory parameters of HCV patients before and after DAA therapy (alanine aminotransferase (ALT), aspartate aminotransferase (AST), high-density lipoprotein (HDL), international normalized ratio (INR), low-density lipoprotein (LDL), model of end-stage liver disease (MELD), not significant (ns), number of cells (n)). Serum from two patients could not be collected at 12 weeks of therapy due to logistical reasons. Statistical test used: Wilcoxon test.

Parameter	Baseline (178 Patients)	12 Weeks of Therapy (176 Patients)	*p*-Value
Age years	54 (24–82)	54 (24–82)	ns
Female/male	74/104	74/102	ns
BMI kg/m^2^	25.6 (17.6–41.6)	25.6 (17.6–41.6)	ns
MELD Score	7 (6–21)	7 (6–21)	ns
Ferritin ng/mL	128.6 (5.6–2309)	94.2 (2.9–1161)	0.004
ALT U/L	61 (2–305)	26 (6–388)	<0.001
AST U/L	47 (7–1230)	22 (6–836)	<0.001
Bilirubin mg/dL	1.0 (1.0–4.3)	1.0 (1.0–7.5)	ns
INR	1.05 (1.00–2.44)	1.04 (1.00–2.22)	ns
Creatinine mg/dL	0.78 (0.14–14.00)	0.76 (0.14–14.7)	ns
Thrombocytes n × 10^9^/L	195 (38–402)	206 (37–407)	ns
Leukocytes n × 10^9^/L	6.5 (2.2–72.4)	6.8 (2.4–62.9)	ns
C-reactive protein mg/L	2.9 (1.0–55.0)	2.9 (2.9–20.3)	ns
Albumin g/L	38 (2–50)	39 (16–93)	ns
HDL mg/dL	52 (19–111)	50 (13–96)	ns
LDL mg/dL	95 (23–296)	119 (33–251)	0.012

## Data Availability

The datasets generated and/or analyzed during the current study are available from the corresponding author upon request.

## Supplementary Materials

The following supporting information can be downloaded at https://www.mdpi.com/article/10.3390/ijms25158161/s1.
