# Supplementary material for: Serum Phosphatidylcholine Species 32:0 as a Biomarker for Liver Cirrhosis Pre- and Post-Hepatitis C Virus Clearance
Source: Int J Mol Sci. 2024 Jul 26;25(15):8161. doi: 10.3390/ijms25158161 (PMC11311844; doi:10.3390/ijms25158161)
Supplement: Supplementary file 1 [file ijms-25-08161-s001.zip › ijms-3066835-supplementary.pdf]

Serum Phosphatidylcholine Species 32:0 as a Biomarker for Liver Cirrhosis Pre- and Post-Hepatitis C Virus Clearance

Kilian Weigand [1,2], Georg Peschel [1,3], Jonathan Grimm [1], Marcus Höring [4], Sabrina Krautbauer [4], Gerhard Liebisch [4] Martina Müller [1] and Christa Buechler [1,*]

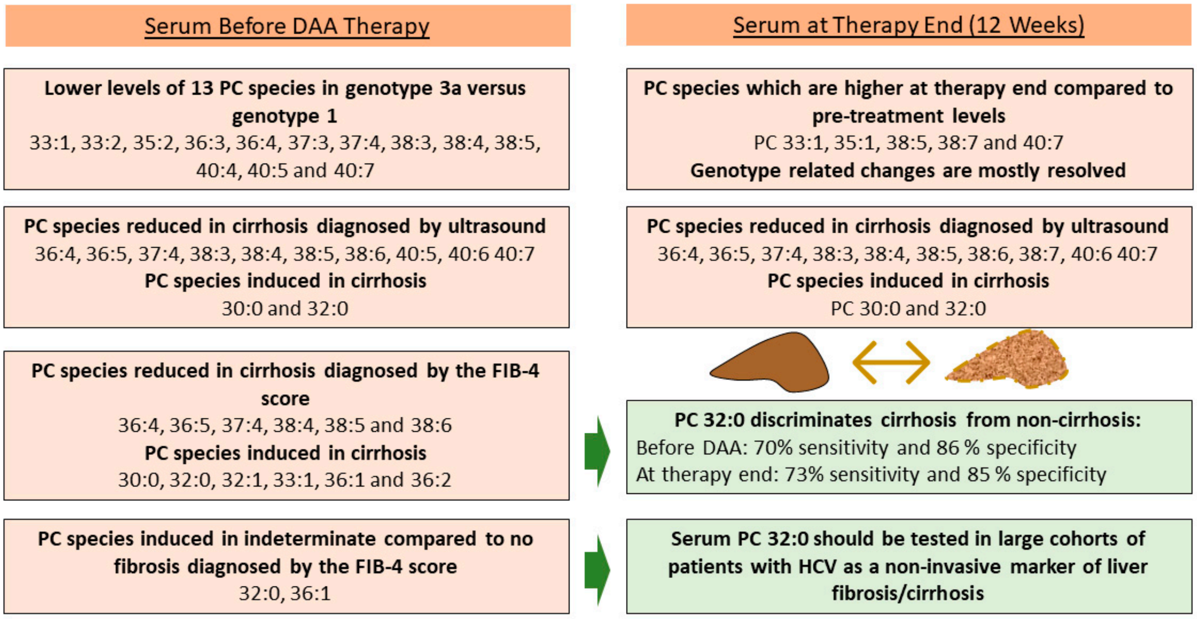

Figure S1. A summary of the main results of our study.

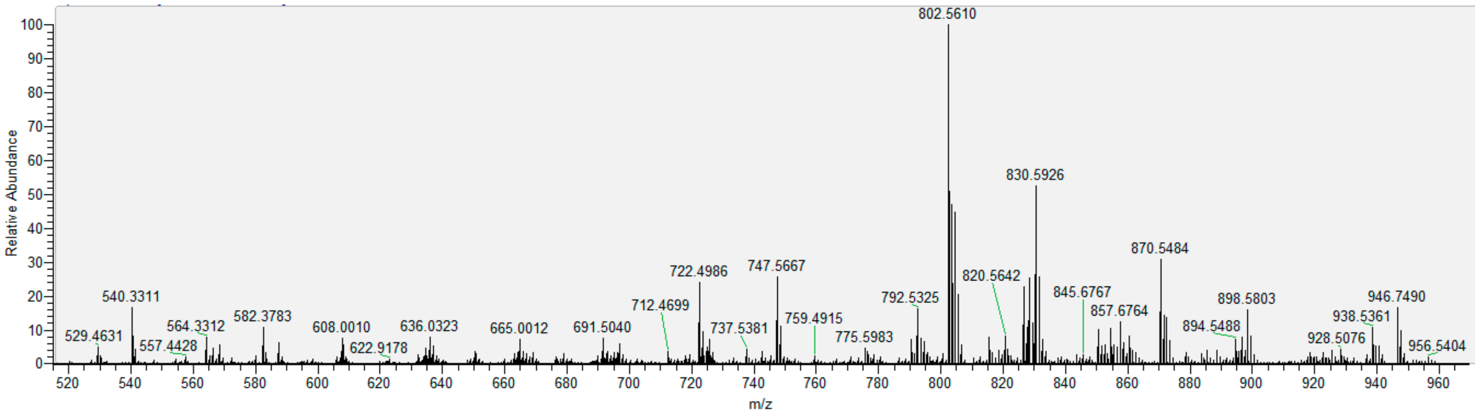

Figure S2. An exemplary full-scan mass spectrum recorded in the negative ion mode in *m/z* range 520-960. Blood of this male patient, who was 55 years old and had liver cirrhosis, was collected at therapy end.
